# The Cost of Caring for the Sick Poor of Atlanta

**Published:** 1887-07

**Authors:** 


### THE COST OF CARING FOR THE SICK-POOR OF ATLANTA.

---

In looking over the annual reports of the city officers, covering the acts and expenditures in their several departments, we are very much impressed by the facts and estimates submitted by Rev. H. H. Tucker, D. D., as President of the Atlanta Hospital and Benevolent Home.

We quote the following from that report. Dr. Tucker says:

"During the year just closed we have afforded board, washing, lodging, fuel, lights, attendance and nursing, when necessary, to two hundred and fifty persons, whose names appear on our register. Besides this we have furnished 186 meals and 67 nights' lodging to persons whose names do not appear, as it is not our custom to record the names of persons who stay with us only one day or less. Taken altogether, we have furnished 11,887 days' entertainment, which is equal to the support of one person for 32 years and 207 days.

"For this service the city has paid us $2,000, which is at the rate of about 16¾ cents per day; expressed in figures, and more accurately, the amount is 16 33–40 cents per day. We have had a larger number of inmates than we ever had before; we have afforded more days' entertainment than we ever did before, and our expense per diem per capita has been less than it ever was before. One fact is worthy of notice: The number of meals and lodgings furnished to transient persons is much less than it has usually been.

"Our table has been abundantly supplied with wholesome food, well prepared and in good variety. The sick have been supplied with luxuries when the condition has required it. So far as we know or believe, no one of our inmates has ever suffered for want of any article of diet."

All thinking people must be gratified by a report which shows that the poor of the city have been provided with board, lodging, fuel, lights, attendance and nursing, when necessary, at the rate of 16¾ cents per day for each person. Every one will feel that the appropriation of $2,000 made to this hospital has been wisely expended. The report shows that for this sum 11,887 days' entertainment was furnished, which, as the distinguished President declares, is equal to the support of one person for thirty-two years and 207 days.

Dr. Tucker asserts that the table has been abundantly supplied with wholesome and well-prepared food in good variety, and that luxuries were furnished the sick when their condition required it, and that no one of the inmates has ever suffered for want of any article of diet.

A more satisfactory report has never been submitted. This is an exhibition of honest and well-ordered economy which must impress every one.

We learn from the Clerk of the City Council that to St. Joseph's Infirmary the city pays for each white person cared for the sum of one dollar per day, and to Ivy Street Hospital the sum of seventy-five cents per day for each white person entertained.

The difference between 16¾ cents and $1.00 in the case of St. Joseph's Infirmary and seventy-five cents paid to Ivy Street Hospital makes an impression on the senses which causes the inquiry: Why these differences? Why should the care of our poor require so much more at one hospital than at another?

The cost of providing for a white pauper of the city is six times as much at St. Joseph's Infirmary as at the Atlanta Hospital, where, we are informed by Dr. Tucker, everything necessary is furnished in abundance and of good quality at 16¾ cents per day.

The cost at Ivy Street Hospital is about four times as great as the charge for the same service at the Atlanta Hospital.

Instead of providing for 250 persons, at a cost of $2,000.00, as was done by the last mentioned, hospital, it would require, at the present rate of payment, about $12,000.00 at St. Joseph's Infirmary and about $8,000.00 at the Ivy Street Hospital.

This comparison very clearly indicates unnecessary expenditure of the public money for the care of the sick-poor of Atlanta. If it was necessary to pay seventy-five cents or one dollar per day for each person in order to secure them reasonable comfort, we would say let this be done. But when the fact that 16¾ cents per day is ample for this purpose comes to us in the report of such a man as the President of the Atlanta Hospital and Benevolent Home, we are constrained to call for legitimate reform.

We learn from official sources that the sum of $1,500.00 per annum is appropriated by the City Council to the Ivy Street Hospital to pay for the care given by that institution to colored paupers, with an additional $600.00 to pay for medicines, and that these amounts are paid without regard to the number so provided for.

We dislike to criticise the acts or contracts of men in whose good judgment and integrity the public have full confidence, but in view of this appropriation we must suggest that a better method of compensation could and ought to be adopted. By appropriating so much per day per capita, both the city and the hospital would be saved from the contingency of loss. Suppose that only ten colored persons should be sent to this hospital during the year, would not the appropriation be extravagant? On the other hand, if 500 should be sent to this hospital during the year, would this sum not fall below such compensation as would be equitable? This method is uncertain and is likely to prove harmful in every instance to either the city or the hospital.

We think Atlanta ought to have her own hospital, and that it should be independent of all medical colleges, as well as every other private interest or enterprise. Such an hospital should be under the sole management of a board of trustees of her best citizens.

If this policy cannot be enforced now, we think it is of prime importance that the expenses of keeping the poor of the city at the several hospitals should be made equal on the basis for estimates furnished by Dr. Tucker in his admirable and gratifying report.

